# Porous metallosilicates for heterogeneous, liquid-phase catalysis: perspectives and pertaining challenges

**DOI:** 10.1098/rsos.171315

**Published:** 2018-02-07

**Authors:** Ceri Hammond, Daniele Padovan, Giulia Tarantino

**Affiliations:** Cardiff Catalysis Institute, Cardiff University, Park Place, Cardiff CF10 3AT, UK

**Keywords:** zeolites, catalysis, green chemistry

## Abstract

Porous silicates containing dilute amounts of tri-, tetra- and penta-valent metal sites, such as TS-1, Sn-β and Fe-ZSM-5, have recently emerged as state of the art catalysts for a variety of sustainable chemical transformations. In contrast with their aluminosilicate cousins, which are widely employed throughout the refinery industry for gas-phase catalytic transformations, such metallosilicates have exhibited unprecedented levels of performance for a variety of liquid-phase catalytic processes, including the conversion of biomass to chemicals, and sustainable oxidation technologies with H_2_O_2_. However, despite their unique levels of performance for these new types of chemical transformations, increased utilization of these promising materials is complicated by several factors. For example, their utilization in a liquid, and often polar, medium hinders process intensification (scale-up, catalyst deactivation). Moreover, such materials do not generally exhibit the active-site homogeneity of conventional aluminosilicates, and they typically possess a wide variety of active-site ensembles, only some of which may be directly involved in the catalytic chemistry of interest. Consequently, mechanistic understanding of these catalysts remains relatively low, and competitive reactions are commonly observed. Accordingly, unified approaches towards developing more active, selective and stable porous metallosilicates have not yet been achieved. Drawing on some of the most recent literature in the field, the purpose of this mini review is both to highlight the breakthroughs made with regard to the use of porous metallosilicates as heterogeneous catalysts for liquid-phase processing, and to highlight the pertaining challenges that we, and others, aim to overcome during the forthcoming years.

## 1. An introduction to metallosilicates

### 1.1. Metallosilicates

**Figure 1.**
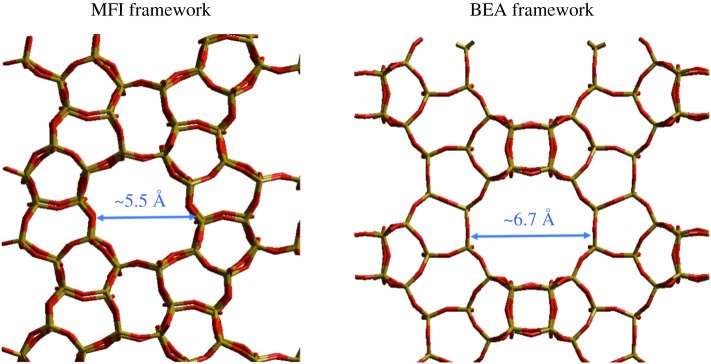
Structure of MFI- and BEA-type zeolites, possessing 10- and 12-membered main rings, respectively. Reproduced with permission from Hammond [3].

Metallosilicates, more commonly known as zeolites, are microporous, crystalline silicate materials composed of corner-sharing TO_4_ tetrahedra. In their conventional form, T represents Si and Al, and the materials are known as aluminosilicates. In such materials, neighbouring SiO_4_ and AlO_4_^–^ tetrahedra are bridged by oxygen atoms, and are regularly arranged into a three-dimensional system of cages and pores with dimensions of 3–20 Å [1–3]. Although there is a diverse number of pore sizes, a critical feature of the pore system of an individual zeolite is its uniformity. Consequently, metallosilicates are readily able to discriminate between molecules with dimensions greater than 0.1 Å, giving rise to their ‘molecular sieving’ ability. This property is key to their use as catalysts and membranes. Two well-known zeolite structures, mordenite framework inverted (MFI) and BEA, are represented in figure 1. As can be seen, whereas molecules less than 6.7 Å would be able to diffuse through the BEA framework, molecules above 5.5 Å would not be able to diffuse through the MFI framework.

**Figure 2.**
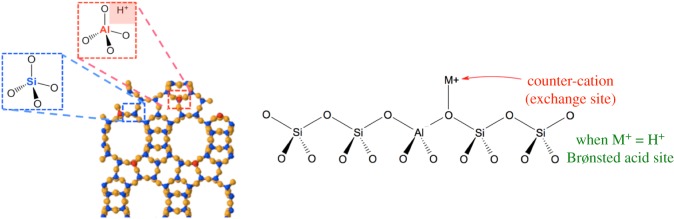
Topological representation of zeolite materials, and how Brønsted acidity arises due to the incorporation of trivalent metals such as Al^3+^ into the framework. Reproduced with permission from Hammond [3].

Whereas totally siliceous materials are highly crystalline polymorphs of silica, and are therefore uncharged, the incorporation of the AlO_4_^−^ tetrahedron into the structures leads to materials with an overall negative charge. To maintain electroneutrality of the framework, an associated ‘extra-framework’ cation, such as NH_4_^+^ and H^+^ is subsequently required. This cation is exchangeable, and gives zeolites wide application as ion-exchange materials, in fields such as water purification. When the cation is a proton (H^+^), the material displays high levels of Brønsted acidity, with acid strength comparable to 100% H_2_SO_4_ (figure 2) [4]. This Brønsted acidity is the basis to the catalytic activity displayed by zeolites for a number of traditional refinery reactions, such as hydrocarbon cracking and isomerization [5]. The number of cation sites is proportional to the AlO_4_^−^ content, and is thus related to the Si/Al ratio of the structure, which can vary from 1 to ∞ [5,6]. By contrast, the strength of each proton site is inversely proportional to the Si/Al ratio, and decreases with increasing Al content. As such, the overall Brønsted acidic properties of any particular zeolite are a combination of both its site strength and density, and hence its Si/Al ratio. Notably, increasing the Si/Al ratio also increases the hydrothermal stability and hydrophobicity of a zeolite [5,6]. The hydrophobicity of a zeolite is thought to be a critical property towards the unique levels of performance demonstrated by new metallosilicates for liquid-phase catalysis.

In addition to Brønsted acidity, conventional aluminosilicates can also display Lewis acidity through ‘dealumination’ of the framework. In this instance, high-temperature treatments, occasionally in steam or other non-inert chemical atmospheres, dislodge Al^3+^ from the framework, leading to the formation of ‘extra-framework’ Lewis acid sites, within the pores of the zeolite. However, it should be noted that the generation of Lewis acid sites through dealumination corresponds to the loss of the cation-exchange sites of the zeolite, and hence diminishes its Brønsted acidity. It also typically results in a material possessing both Lewis and Brønsted acidity, because total dislodgement of Al^3+^ from the framework is highly challenging, and some residual framework Al^3+^ typically remain. Moreover, it can result in the generation of a defective framework, which may impact its (hydro)thermal stability. Accordingly, such materials are rarely uniform, and often display disparate levels of activity, selectivity and/or stability during catalysis.

### 1.2. Conventional applications of zeolite catalysts

Zeolites possess several properties that make them ideal materials for a variety of catalytic and technological applications. One is their molecular sieving ability, a feature that zeolites share with enzymes [7,8]. This property is mainly exploited in separation and purification technologies, but can also be used to influence the chemo- and/or regio-selectivity of catalytic processes through ‘shape selectivity’ [9,10], or induce reactivity through confinement effects [11,12]. Their tuneable acid–base properties also make such materials highly desirable. Indeed, H^+^-form zeolites, formed by inclusion of the AlO_4_^−^ tetrahedron, efficiently catalyse traditional Brønsted acid-catalysed reactions, such as cracking or isomerization. Aluminosilicates can also act as Lewis acids in chemical reactions such as Friedel–Crafts (FC) alkylations, following the generation of extra-framework Al^3+^ sites by dealumination [6,13]. It is worth repeating here, however, that occluding Al^3+^ into the extra-framework results in a material containing Lewis and residual Brønsted acidity. Although effective as catalysts, such ill-defined materials make precise elucidation of the catalytic mechanism highly challenging, leading to inefficient, trial-and-error-based catalyst improvement methodologies. Moreover, their residual Brønsted acidity can dramatically influence the selectivity of a catalytic process, and their defective nature also leaves them unstable towards (hydro)thermal dissolution, especially in liquid media. Consequently, the development of analogue materials, possessing Lewis acidity and/or redox ability alone, along with a high degree of hydrothermal stability, is of critical importance.

## 2. State of the art: liquid-phase processes catalysed by porous metallosilicates

### 2.1. Emerging Al^3+^-free metallosilicate materials

As the chemical industry aims to become more sustainable, the use of renewable feedstock is becoming increasingly relevant. Given that such feedstock, such as sugars, are often highly oxygenated, and hence possess low levels of vapour pressure and thermal stability, conventional methods of upgrading, i.e. gas–solid, are rarely suitable. As such, a shift towards low temperature, liquid-phase catalytic chemistry is essential. Even for conventional catalytic processes, such as selective oxidations, the development of liquid-phase methods can also offer improved process efficiency, through decreased energy demands and/or improved processing selectivity. Consequently, the developments of catalysts exhibiting high levels of activity even in a liquid medium are required.

Along with typical aluminosilicates, analogous structures containing other main group and transition-metal elements, including Ti, Sn, Ge, Ga, B, Fe, Co, Mn and P, are also known [14–16]. In contrast with aluminosilicates, the isomorphous substitution of tetravalent species such as Sn^4+^ and Ti^4+^ into the structure results in the generation of Lewis acid sites without the co-generation of Brønsted acid sites [14–16]. Furthermore, inclusion of a suitable transition metal into the structure, e.g. Fe^3+^, can also endow the zeolite with redox properties, in addition to acid–base capability [17]. As such, zeolites may behave independently as Brønsted and Lewis acids, bifunctional Brønsted/Lewis acid catalysts or as redox active catalysts, depending on the identity and the location of the heteroatom. The following section therefore outlines some of the most novel uses of porous metallosilicates, and highlights how subtle modifications to the composition and/or structure of a zeolite can dramatically influence its properties and reactivity patterns. Particular emphasis is placed on Lewis acidic and/or redox active metallosilicates that catalyse emerging liquid-phase applications.

### 2.2. Titanium-containing silicates: a revolution in liquid-phase oxidations

The discovery and development of titanium silicalite-1 (TS-1) by researchers at EniChem represents a milestone in zeolite science [14,18]. TS-1 is a porous metallosilicate within which a small amount of silicon atoms is substituted for tetravalent Ti^4+^. TS-1 is an example of an MFI-type zeolite (MFI = Mordenite framework inverted), which possesses two 10-ring tunnel systems, one straight (5.4 × 5.6 Å) and one sinusoidal (5.1 × 5.5 Å) (figure 1). Consequently, the material is suitable for catalytic applications involving small-to-medium-sized substrates. Unlike conventional aluminosilicates, TS-1 is exclusively Lewis acidic, and is therefore able to selectively perform Lewis acid catalysis [18]. Moreover, whereas Lewis acidic aluminosilicates are typically hydrophilic, due to the residual charge of the lattice or the presence of defect sites following dealumination, the Lewis acidic sites present in TS-1 are encapsulated within a highly hydrophobic, defect-free siliceous architecture. Therefore, unlike conventional Lewis acid catalysts, TS-1 is able to act as a Lewis acid catalyst even in polar solvents, such as methanol and water, because active-site hydrolysis, oligomerization and decomposition is hindered [18,19]. The presence of a hydrophobic, non-charged lattice also makes TS-1 and its analogues ideal catalysts for the oxidation of hydrophobic species such as alkanes, because the partially oxygenated products produced during the reaction are preferentially diffused out of the zeolite pores, minimizing their potential consecutive oxidation.

**Figure 3.**
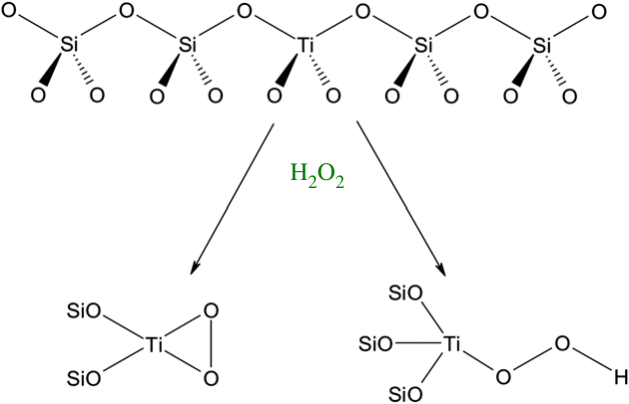
Simplified representation of TS-1 and the potential oxidative species formed through its interaction with hydrogen peroxide. Reproduced with permission from Hammond [3].

Owing to these unique properties, TS-1 displays unique reactivity patterns. It is now generally accepted that isomorphously substituted Ti^4+^ atoms in the lattice are responsible for catalytic performance. The Ti^4+^ sites are proposed to be tetrahedral when dehydrated, but can expand their coordination number to six following interaction with solvent molecules or reactants. The Lewis acidic nature of TS-1 arises from the d^0^ character of Ti^4+^, which enables it to form acid–base adducts with various reactant species. Although this can be used to catalyse a variety of Lewis acid-type reactions, such as isomerization and coupling reactions, the unique reactivity of TS-1 is best exploited when it reacts at mild conditions with hydrogen peroxide (H_2_O_2_). During this, a variety of metal-peroxo and/or metal-hydroperoxo species can be formed (figure 3), although the true identity of these intermediates remains the topic of much debate [20]. After molecular oxygen and air, H_2_O_2_ is perhaps the most sustainable oxidant in the chemical industry, given its high quantity of active oxygen (47%), and the relatively benign nature of its reduced form, H_2_O.

**Figure 4.**
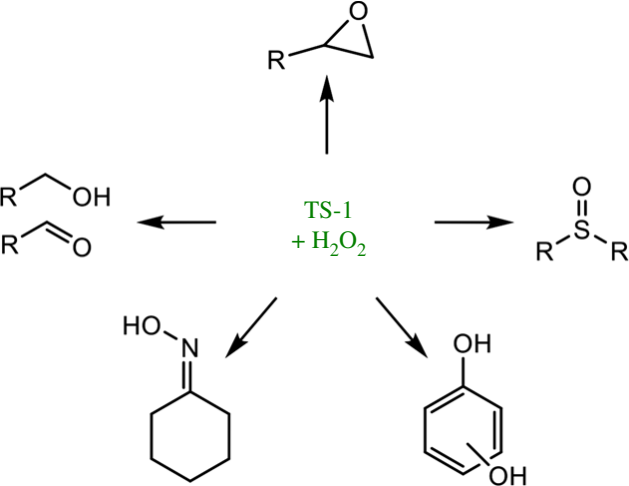
Simplified scheme demonstrating some of the catalytic oxidations mediated by TS-1 and H_2_O_2_. Reproduced with permission from Hammond [3].

Since the discovery of its unique ability to activate H_2_O_2_, several innovative technologies have been developed with TS-1 as heterogeneous catalyst. The most notable is the recently commercialized ‘hydrogen peroxide to propylene oxide’ (HPPO) process, during which TS-1 and H_2_O_2_ interact to convert propylene into propylene oxide, a key platform molecule in the bulk chemical industry (8 million tonnes per annum) [21,22]. In addition to decoupling propylene oxide production from other value chains, the HPPO process is also characterized by an improved environmental and economic footprint relative to other production routes, such as the chlorohydrine process and the `styrene monomer propylene oxide' (SMPO) process. Indeed, eco-efficiency analysis reveals that the HPPO process reduces wastewater production by over 70%, energy use by 35% and capital investment by 25%. In addition to the HPPO process, TS-1 also exhibits unique levels of activity for several other oxidation processes (figure 4), including the catalytic ammoxidation of cyclohexanone to cyclohexanone oxime, an important precursor to caprolactam and Nylon 6, and the hydroxylation of phenol to catechols and quinones. The development of TS-1 clearly exemplifies how the development of a novel catalytic material, possessing unique physical and chemical properties, provides opportunities to revolutionize the chemical value chain. In fact, TS-1 remains the only Lewis acidic silicate capable of selectively catalysing several of these catalytic processes, despite analogous silicates containing other Lewis acidic elements, such as Sn^4+^, being developed. More recently, analogous Ti-based silicates with larger pore sizes, such as Ti-β and Ti-MWW, have also been developed [23,24]. Such materials improve the general applicability of Ti-based silicates beyond small molecules, and improve their resistance to fouling and transport limitations.

### 2.3. Stannosilicate zeolites as liquid-phase catalysts

**Figure 5.**
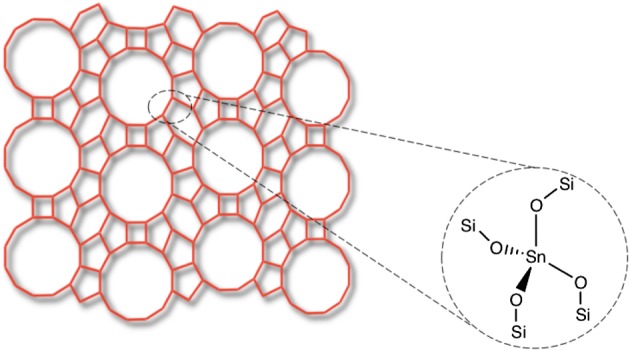
Simplified structural representation of Sn-containing zeolite *β*. Reproduced with permission from Hammond [3].

Sn-β is another example of a purely Lewis acidic zeolite, in which small amounts of Sn^4+^ are isomorphously substituted into the zeolite framework (figure 5). However, while TS-1 is highly active and highly selective for a variety of oxidations with H_2_O_2_ [24], Sn-containing zeolites are typically more active and selective for reactions involving oxygenated reactants, such as biomass-derived glucose or various ketone-based conversions (figure 5) [19,25–28].

**Figure 6.**
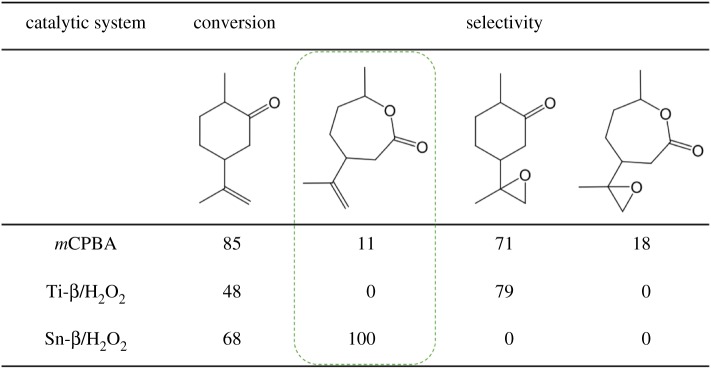
Unique chemoselectivity exhibited by Sn-β/H_2_O_2_ during the Baeyer–Villiger oxidation of substrates possessing multiple functional groups. The desired BVO product is highlighted in the dashed green box. Reproduced with permission from Hammond [3].

**Figure 7.**
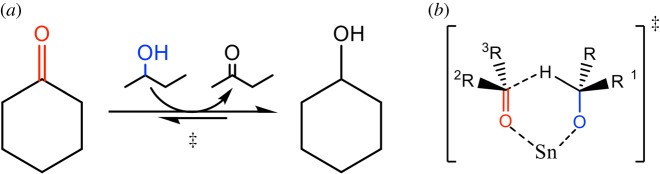
Meerwein–Ponndorf–Verley transfer hydrogenation mediated by Sn-β. (*a*) Overall reaction scheme, and (*b*) six-membered transition state responsible for hydride transfer involving the activated carbonyl group and the deprotonated alcohol. Reproduced with permission from Hammond [3].

First discovered in 1996 [29], interest in Sn-β surged following the seminal report of the Corma group, who demonstrated that Sn-β is a uniquely active and selective catalyst for the Baeyer–Villiger oxidation (BVO) with H_2_O_2_ [15]. BVO is a very favourable process to convert ketones into valuable esters and lactones, and is widely employed in the bulk and fine chemical industries. On a bulk scale, a major example of BVO is the conversion of cyclohexanone to ϵ-caprolactone, a key precursor in several polymer processes. Typically performed with oxidants such as peracetic acid, it was shown by Corma *et al.* that unprecedented levels of activity and selectivity to ϵ-caprolactone could be achieved when Sn-β and H_2_O_2_ were employed as catalyst and oxidant, respectively. In addition to replacing an oxidant characterized by poor atom efficiency, low chemoselective and explosiveness [30], the combination of Sn-β and H_2_O_2_ allows 100% lactone selectivity to be achieved even for highly functionalized substrates (figure 6). To date, no other catalyst has been shown to catalyse this reaction at such high levels of activity and chemo-selectivity. Indeed, whereas Sn-β is exclusively selective to the desired lactone product during the BVO of dihydrocarvone, Ti-β primarily yields the epoxidized product at 79% selectivity (figure 7). While the reason(s) for this disparate performance have not yet been truly identified, it has been hypothesized that it relates to the differing modes of operation of these two analogous catalysts; while Ti-β activates H_2_O_2_ to yield various Ti-(hydro)peroxo species, resulting in both BVO and epoxidation being observed (figure 6), Sn-β reportedly only activates the carbonyl group on the reactant, simply making the substrate more prone to nucleophilic attack [14,31]. This further emphasizes that discrete changes in the composition of the material, such as substituting Ti^4+^ for Sn^4+^, can dramatically influence catalytic performance.

Sn-β has also been shown to be an excellent heterogeneous catalyst for the Meerwein–Ponndorf–Verley (MPV) reaction, more commonly known as catalytic transfer hydrogenation (CTH) [32,33]. In this reaction, co-activation of the carbonyl group of the substrate and the solvent of the reaction facilitates hydride transfer through a six-membered transition state (figure 7). This process permits selective hydrogenation of the carbonyl group to occur even in the absence of high-pressure molecular H_2_, at high levels of activity (greater than 95% conversion) and selectivity (99.5%). Although Ti- and Al-containing *β* zeolites also display activity for the reaction, they have been found to be substantially less active and selective than the Sn-containing material, further demonstrating that not all Lewis acidic zeolites perform equally for different reactions [34].

**Figure 8.**
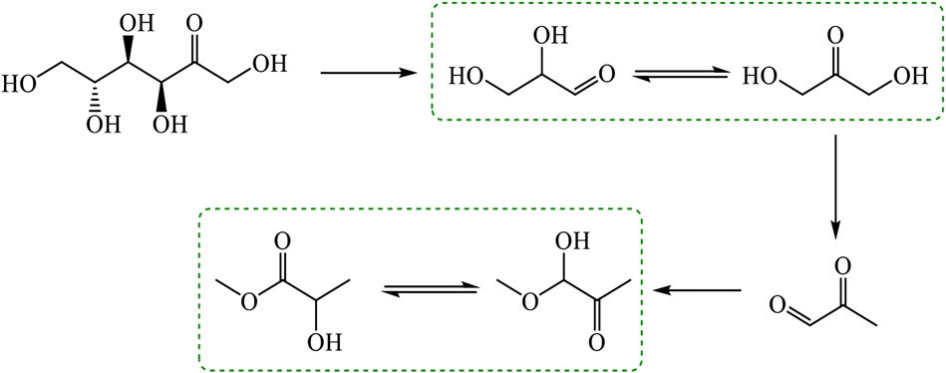
Catalytic conversion of glucose to methyl lactate. The reaction steps catalysed by Sn-β are highlighted by the dashed boxes. Reproduced with permission from Hammond [3].

The ability of Sn-β to activate carbonyl compounds has also been exploited to convert biomass to chemicals and fuels. Indeed, an explosion of research has recently emerged focused upon the utilization of Sn-β as a heterogeneous catalyst for biomass valorization. In 2009, Taarning and co-workers first reported on the ability of Sn-β to catalyse the conversion of small sugar-based compounds, such as dihydroxyacetone and glyceraldehyde, into methyl lactate [35]. Methyl lactate is the methylated ester of lactic acid, the monomer building block of polylactic acid (PLA), a bio-degradable and bio-renewable polymer with several industrial applications. The role of Sn-β, in this case, was to mediate the isomerization of dihydroxyacetone to glyceraldehyde via intramolecular 1,2-hydride transfer, which is the key step in the cascade of reactions and processes that eventually convert bio-renewable compounds into methyl lactate at yields up to 99% at 80°C (figure 8). Once more, the different levels of performance for different Lewis acidic centres was observed, as under the same reaction conditions, Ti-β produced methyl lactate at yields of less than 5%. More recently, the process has been extended so that more abundant and more favourable C_5_- and C_6_-based sugars could be employed as substrates, albeit with slightly lower yields being observed (maximum methyl lactate yield of 68%) [36].

Sn-β has also shown an unprecedented ability to mediate the isomerization of glucose to fructose [37]. Glucose–fructose isomerization is a key step in the production of high-fructose corn syrup (HFCS), and is one of the largest examples of a biocatalytic process (approx. 8 million tonnes per annum), where an immobilized enzyme converts glucose into an equilibrium mixture of unconverted glucose (50 wt%), fructose (42 wt%) and other saccharides (8 wt%) [38]. Given that the biocatalytic process suffers from several disadvantages, particularly in terms of operational flexibility (pH, temperature, solvent) and intensification, the development of an active, robust and versatile heterogeneous catalyst represents a breakthrough achievement. Through a variety of methods, Davis *et al.* demonstrated that Sn-β efficiently catalyses the isomerization process via an intramolecular hydride shift [39], analogous to that observed during CTH and the conversion of triose sugars to methyl lactate. In a similar manner to TS-1, the hydrophobicity of Sn-β allows the use of both water and methanol as reaction media; although isomerization rates were more recently found to be 1–2 orders of magnitude lower in water than in methanol [40], the improvement in glucose solubility can offer a major advantage in terms of intensification. More recently, Sn-β has also been employed to catalyse the formation of so-called ‘rare’, i.e. non-naturally occurring, sugars through epimerization, following ion-exchange with Na^+^ or with borate salts or following [41,42].

**Figure 9.**
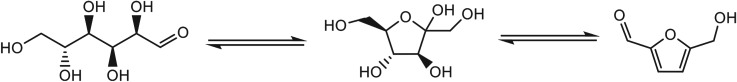
Catalytic production of bio-renewable furanic molecules mediated via glucose–fructose isomerization over Sn-β. Reproduced with permission from Hammond [3].

The isomerization of glucose to fructose also opens up pathways for the conversion of biomass to furanic compounds, given that the dehydration of fructose is more facile than the dehydration of glucose (figure 9) [43]. Such furanic compounds are important target molecules for the chemical industry, as they possess a variety of potential downstream examples [44]. For example, renewable 2,5-furandicarboxylic acid (FDCA) is a potential replacement for terephthalic acid, the key constituent of polyethylene terephthalate (PET) [45,46]. To date, the optimal method of producing high yields of 5-HMF from biomass involves the use of hybrid catalytic systems, whereby a Lewis acidic silicate, such as Sn-β, is employed in conjunction with a homogeneous or heterogeneous Brønsted acid catalyst. In such systems, the Lewis acid catalyses the initial isomerization process, and the Brønsted acid subsequently catalyses the consecutive dehydration reaction [47–49]. Several other cascade processes have since been reported, further demonstrating the versatility of Sn-β and its analogues to upgrade bio-renewable substrates [50–52].

### 2.4. Other Lewis acidic silicates

Other tetravalent Lewis acids, such as Zr^4+^ and Hf^4+^, have also been efficiently incorporated into porous metallosilicate frameworks [25]. In addition to demonstrating the versatility of porous silicates to host a variety of active sites, Zr-β and Hf-β further emphasize how discrete changes to the composition of porous metallosilicates can lead to dramatic changes in reactivity patterns. For example, Wang *et al.* demonstrated that Lewis acidic Zr- and Hf-containing *β* zeolites, were up to four times more active and selective than the typical Sn-containing analogue for the self-aldol condensation of ethyl pyruvate [27]. The unique reactivity of these materials was associated with their ability to catalyse self-condensation through soft enolization, in addition to their ability to tolerate various carboxylic acid groups and water. In addition to high levels of activity, these silicates were also shown to possess good levels of stability, displaying less than 20% loss of activity over a continuous 24 h period (see §3.3). Hf-containing zeolites were also demonstrated by the same group to be exceptionally active catalysts for the cascade conversion of 5-HMF to useful fuel additives, such as alkoxymethyl furans like 2,5-bis(ethoxymethyl)furan [53]. Other pentavalent metal centres, such as Nb^5+^ and Ta^5+^ centres, have also recently been incorporated into the porous silicates via conventional hydrothermal synthesis [25]. Although the precise coordination of pentavalent metals within the structure differs dramatically from that of conventional tri- and tetra-valent species, the Lewis acidity of these centres was shown to be sufficient to catalyse CTH and etherification reactions [25]. Ta^5+^-containing zeolites were also recently shown to be active catalysts for the conversion of ethanol to 1,2-butadiene, a key route towards converting bio-renewable ethanol into important bulk chemicals [54].

### 2.5. Porous metallosilicates containing redox-active centres

In contrast with porous metallosilicates containing non-redox active species, the ability of centres such as Fe^3+^ to participate in redox chemistry, in addition to acid–base catalysis, further influences the reactivity of these materials. Among such materials, Cu^2+^- and/or Fe^3+^-containing ZSM-5 are best known, due to their ability to catalyse several gas-phase processes [17], including the decomposition of nitrous oxide (N_2_O decomposition), the selective reduction of various nitrous oxides (SCR reaction), and hydroxylate benzene to phenol [55]. Indeed, studies concerning the catalytic activity of Fe-ZSM-5 for gas-phase oxidation reactions were reported as early as 1988, when three independent research groups discovered the ability of this material to convert benzene into phenol with N_2_O as terminal oxidant [56–58]. Along with providing a more atom-efficient and selective route to phenol, the one-step process with N_2_O and Fe-ZSM-5 also decouples phenol production from acetone, which is typically co-produced on a 1 : 1 molar ratio during the standard process [59]. A vast number of studies in the intervening years have since demonstrated that the same system is capable of activating the C-H bonds of methane, albeit in a non-catalytic manner (TON < 1) [60].

**Figure 10.**
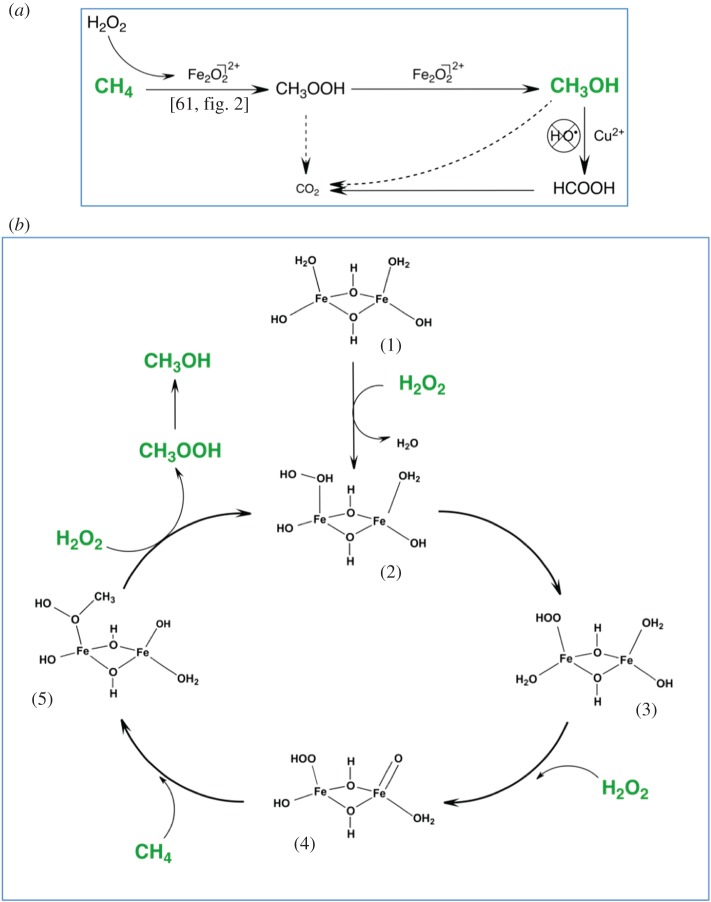
Catalytic conversion of methane to methanol following the activation of H_2_O_2_ by Fe-containing ZSM-5-type zeolites. (*a*) Overall reaction network. (*b*) Detailed mechanism of H_2_O_2_ and CH_4_ activation by dimeric Fe^3+^ species. Reproduced with permission from Hammond *et al.* [61].

More recently, it has been shown that such materials can also be employed as heterogeneous catalysts for liquid-phase catalysis. For example, some of us recently demonstrated that Fe-containing zeolites were able to selectively convert methane into methanol via the catalytic activation of H_2_O_2_ [61]. Unlike previously reported gas-phase systems, the liquid-phase system involving Fe-ZSM-5 and H_2_O_2_ is both catalytic (TON numbers greater than 10 000 observed after five successive catalytic cycles) and uniquely selective to the desired product, methanol (up to 95%), even at high levels of methane conversion [62]. By contrast, the gas-phase systems yield methanol at sub-stoichiometric quantities (TON < 1) and involve destructive solvothermal treatments of the catalyst to liberate this small amount of product. The liquid-phase system is also shown to be suitable for operation at mild non-acidic conditions (2–70°C), similar to those employed by the sMMO enzyme. The key to this unique reactivity is the heterolytic activation of H_2_O_2_ by Fe^3+^ species within the zeolitic micropores. In such a case, the Fe^3+^ sites act primarily as Lewis acid sites, probably resulting in the formation of various ferric (III) hydroperoxide species, analogous to the Ti^4+^ centres in TS-1. In contrast with Ti^4+^, however, the redox activity of Fe^3+^ allows it to further react with the coordinated peroxide moiety. Indeed, through various spectroscopic and computational methods, it was proposed that activation of the resting stage of the catalyst, computationally described as a di-μ–hydroxo-bridged iron complex [Fe_2_(μ_2_-OH)_2_(OH)_2_(H_2_O)_2_]^2+^ (species 1 in figure 10) [61], eventually results in the formation of a bifunctional active site possessing both iron-oxo and iron-hydroperoxo moieties (species 4). Such oxo-compounds can evidently only be obtained when the Lewis acid sites present in the catalyst possess higher oxidation states, as is the case for Fe^3+^. Such intermediates are reminiscent of those formed in biological systems, and raises the enticing prospect that this catalytic system operates in a biomimetic peroxide shunt-type process. Nevertheless, *in situ* characterization of the catalyst, and definitive spectroscopic identification of the (short-lived) reactive intermediates in question has yet to be achieved. At present, this precludes further comparison to biological systems, and prohibits further exploitation of this promising heterogeneous catalyst for other reactions of biological and industrial interest. Interestingly, incorporation of Cu^2+^ into the Fe-ZSM-5 material was shown to dramatically influence the selectivity of the reaction, by switching off the consecutive oxidation of methanol to formic acid and CO_x_. However, the role of Cu^2+^ has yet to be truly understood [62].

## 3. Perspectives and pertaining challenges

It is clear that porous metallosilicates have emerged as uniquely active catalysts for a variety of liquid-phase catalytic transformations, including the upgrading of bio-renewable compounds, and various selective oxidation processes. While one of these materials has already reached industrial levels of exploitation (TS-1), many challenges remain to be overcome before several other materials reach similar levels of exploitation. The purpose of the following section, therefore, is to draw attention to several pertaining challenges in this field, and to highlight some of the most recent research studies focused upon overcoming them.

### 3.1. Synthesis of porous metallosilicates, such as Sn-β

Conventional zeolite preparation involves hydrothermal synthesis, during which the basic ingredients for synthesis (silicon and aluminium source, and a structure-directing agent (SDA)) are dissolved in an aqueous solution containing a mineralizing agent. Treatment of the gel under autogenic conditions (high temperature and pressure) in the presence of the structure-directing agent leads to formation of the zeolite powder. Thermal removal of the residual SDA from the pores, following filtration from the remaining sol gel, yields the final porous, crystalline zeolite [63]. The synthesis of porous metallosilicates containing Lewis acidic centres is also achieved via hydrothermal synthesis. However, while several silicates, such as aluminosilicates and titanosilicates, can be crystallized in the presence of basic mineralizing agents, several recent analogues, such as Sn-β, cannot be prepared under such conditions [64–66]. Indeed, while Mal & Ramaswamy were able to crystallize Sn-MFI containing isomorphously substituted Sn sites in the presence of ^–^OH as mineralizing agent [67], crystallization of Sn-β under these conditions could only be achieved when Al^3+^ was co-included into the framework [29].

**Figure 11.**
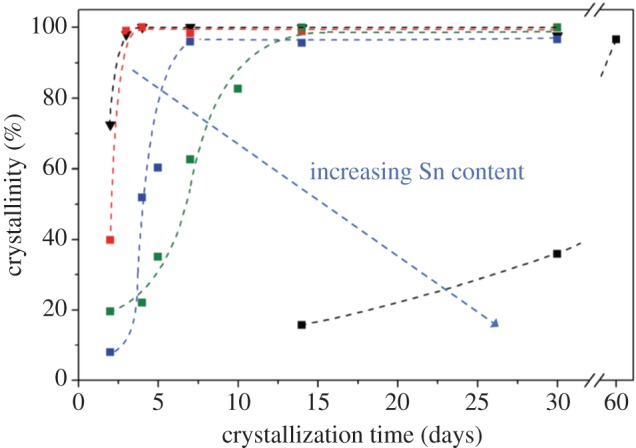
Effect of Sn content and crystallization time on the crystallinity and morphology of Sn-β. Adapted with permission from Tolborg *et al*. [68].

To achieve synthesis of a purely Lewis acidic silicate-containing species such as Sn^4+^, use of F^−^ as mineralizing agent is required [15]. However, while this permits the synthesis of a purely Lewis acidic silicate with several favourable physical properties, including high levels of hydrothermal stability, hydrophobicity and crystallinity, several drawbacks of this method prevail. The first drawback involves the requirement of HF as mineralizing agent. Not only does HF lead to significant health and safety concerns, its corrosiveness means the production instrumentation also needs to be acid-resistant. Furthermore, while crystallization in HF results in a material with some physical advantages, the crystallites obtained by this method are typically very large, especially at higher Sn loadings [68]. Such crystallites can be detrimental to catalytic performance. The formation of inactive SnO_2_ domains on the external surface of the zeolite also appears to be unavoidable by this methodology [69], and crystallization times greater than 40 days are routinely required for sufficient levels of crystallinity to be achieved. Unfortunately, the requirement for long crystallization times arises from the presence of the Sn precursor, with a progressive increase in the Sn content of the gel leading to dramatic increases in the required crystallization time (figure 11) [68]. Another unavoidable limitation of this synthetic approach is the low amount of active metal that can be incorporated, with a maximum limit of 2 wt% Sn being encountered. Accordingly, catalysts prepared by this method have an especially low loading of metal in terms of SiO_2_/SnO_2_ molar ratio, which leads to low levels of catalyst productivity (g product g^−1^ catalyst), and low levels of space–time yield (g (product produced) cm^−3^ (reactor volume) h^−1^) being obtained [70].

Given these disadvantages, the development of more generally applicable and more scalable preparation methodologies for porous metallosilicates represents one of the major pertaining challenges in the field. Among recently explored alternatives, two major types of preparation strategies can be identified, those being ‘bottom up’ and ‘top down’. In the former case, faster and/or more selective methods of constructing these desirable materials through sol-gel methods are targeted. In the latter, the conversion of readily available aluminosilicates into the more desirable porous metallosilicate form is targeted [71]. Although various bottom-up methods exhibit potential [72,73], the greatest breakthroughs with regard to porous metallosilicate synthesis, to date, have been achieved with top-down methods.

**Figure 12.**
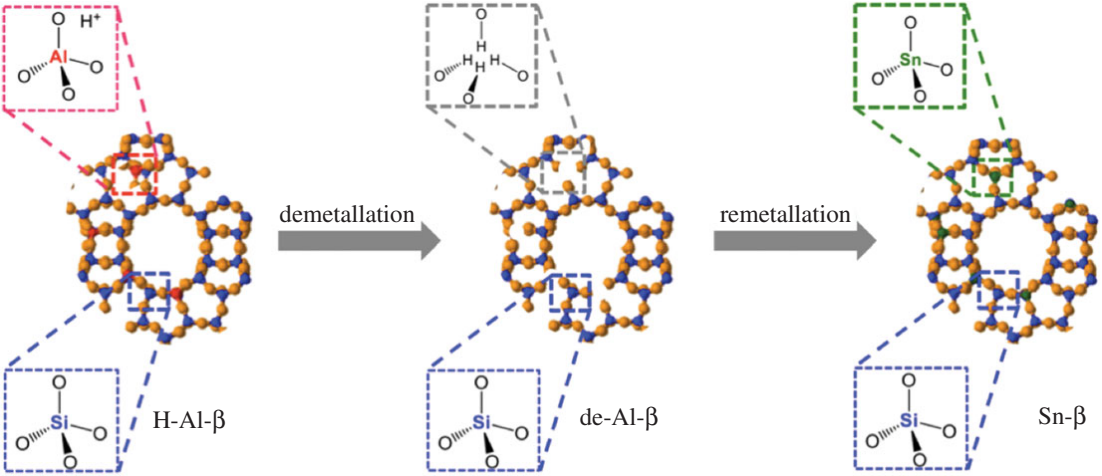
Graphical representation of the synthesis of Sn-β via post-synthetic demetallation–remetallation. Reproduced with permission from Hammond [3].

**Figure 13.**
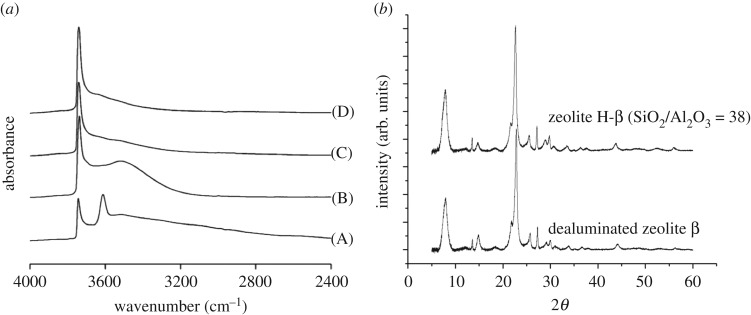
Formation of silanol nests as evidenced by (*a*) FTIR spectroscopy. (*b*) Retention of the BEA framework following dealumination in concentrated HNO_3_ at 100°C. Adapted with permission from Hammond *et al*. [75].

Top-down methods typically convert an easily prepared or commercially available zeolite material into a more desirable form by post-synthetic treatment. The most intensely studied approach in recent years is the demetallation–remetallation strategy (figure 12) [74]. In this method, defect sites are first created by dealumination of an aluminosilicate host matrix, although deborylation and desilication can also be followed. Total dealumination is typically achieved by acidic treatment. For example, we demonstrated that treatment of zeolite *β* in concentrated acidic media, e.g. HNO_3_, 1–13 M, at elevated temperatures up to 100°C results in the formation of an Al^3+^-free framework with retained crystallinity [75]. Removing Al^3+^ from the structure results in the formation of vacant framework sites, commonly known as silanol nests, as observed by ^29^Si NMR and FTIR spectroscopy (figure 13) [75].

Following demetallation, incorporation of the desired heteroatom into the framework is targeted through remetallation, with vapour–solid [76,77], liquid–solid [78–81] and solid–solid [75] methods all having been attempted in recent years. For example, the incorporation of up to 6 wt% Sn into dealuminated zeolite *β* by chemical vapour deposition of SnCl_4_ at 673–773 K was achieved by Li *et al.* [76] and Liu *et al.* [77]. Dijkmans *et al.* on the other hand, were able to incorporate between 0.3 and 8.6 wt% Sn into the vacant sites of zeolite *β* by refluxing the dealuminated material in dry isopropanol containing Sn (IV) chloride pentahydrate for 7 h [78,79]. A liquid–solid approach was also followed by Dapsens *et al.* who used alkaline-assisted metallation (AAM) to functionalize partially desilicated matrices with Lewis acidic centres [80,81]. In contrast with other demetallation–remetallation strategies, Dapsens *et al.* generated vacant framework sites by partial desilication in mild caustic media (0.2 M NaOH, 65°C, 0.5 h) [81]. The mild conditions of desilication can present a major advantage over dealumination, which typically requires harsher conditions (13 M HNO_3_, 100°C, 20 h). Moreover, it also allows structures that are not amenable to dealumination to be functionalized [80,81]. Another advantage of the method is that partial desilication can also present an opportunity to generate hierarchical zeolites [82,83].

On the other hand, our team has demonstrated that solid–solid methods can also be used for the preparation of porous metallosilicates. In our studies, a combination of mechanochemical and thermal treatment of dealuminated zeolite *β* with a suitable metal precursor, such as Sn (II) acetate, results in the inclusion of the desired heteroatom [75,84]. This approach, termed solid-state incorporation (SSI), provides several major advantages over other top-down methods, including rapid rates of synthesis (total time for mechanochemical and heat treatment steps being less than 8 h), the absence of solvents and wet chemicals in the remetallation stage, and the ability to incorporate high loadings of metal (less than 10 wt%). Moreover, the procedure is highly versatile, allowing incorporation of species such as Fe^3+^, Ti^4+^ and Zr^4+^ in catalytically active forms [85,86]. We have demonstrated that the materials produced by SSI are equally as active per mole of active site as their conventionally prepared counterparts for the isomerization of glucose to fructose, BVO and CTH, among others. However, due to their higher metal loadings, their space–time yields are up to one order of magnitude larger [75].

**Figure 14.**
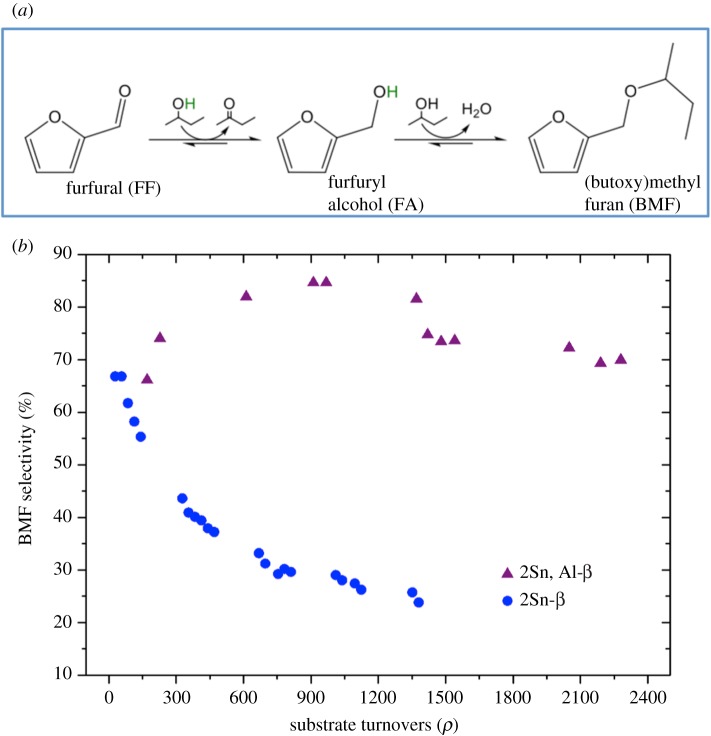
(*a*) Catalytic conversion of furfural to BMF via CTH and *in situ* etherification. (*b*) BMF selectivity as a function of substrate turnovers for Sn-only, and Sn, Al-containing *β* zeolite. Reproduced with permission from Padovan *et al*. [88].

In general, top-down methods possess several advantages over conventional bottom-up methods. The most evident are the rapid rates of synthesis, and the absence of HF from the procedure. Further advantages include the ability to obtain materials with smaller crystallite sizes, and opportunities for increasing the metal loading of the materials beyond those possible through hydrothermal synthesis. A final advantage, as reported by the group of Sels [87] and by our team [88], includes the possibility of obtaining bifunctional catalysts, by combining partial dealumination of the parental zeolite along with remetallation. Although disadvantageous in some cases, such bifunctional materials have been shown to display unique levels of reactivity and selectivity for various reactions, including the conversion of dihydroxyacetone to ethyl lactate, and the production of alkoxymethyl furans from furfural [79]. Indeed, we recently demonstrated that a bifunctional catalyst containing both Sn^4+^ and Al^3+^ was uniquely selective for the conversion of furfural to (butoxy)methyl furan (BMF), a potential bio-renewable fuel additive. The bifunctional catalyst was found to maintain selectivity to BMF above 70% even after 2000 substrate turnovers, whereas the Sn-only catalyst lost almost all its selectivity to BMF after less than 900 turnovers (figure 14) [88].

**Figure 15.**
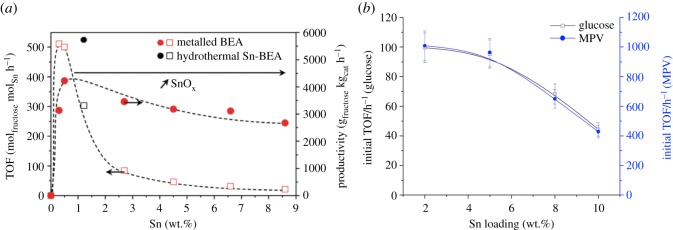
Variation in TOF and productivity of Sn-β at various Sn contents. (*a*) As prepared by liquid–solid methods, and (*b*) as prepared by SSI. Figures reproduced with permission from Dijkmans *et al*. [78] and Hammond *et al*. [84].

From these recent studies, however, it is clear that incorporating high loadings of active species into the demetallated precursor remains a considerable challenge. Indeed, in all these previous studies, a mixed active-site distribution was observed at metal loadings higher than 2 wt%, despite the presence of enough vacant framework sites for up to 10 wt% of Sn. However, the degree to which non-active Sn species form appears to be highly dependent upon the choice of incorporation. For example, whereas Dijkmans *et al.* observed a steady decrease in turnover frequency (TOF) between 0.3 and 8.6 wt% Sn [78], our team observed that comparable TOFs could be obtained up to a maximum loading of 5 wt%, beyond which a decrease in TOF, and hence an increase in the number of spectator species, is observed (figure 15) [84]. Clearly, developing more suitable methods for incorporating higher loadings of heteroatoms into the framework of demetallated zeolites remains an important challenge if sufficient productivity and space–time-yield values are to be obtained. Additionally, it is essential to further optimize the demetallation–remetallation approach so that other silicate structures can also be functionalized in this manner. Finally, elucidating the mechanism of remetallation, and understanding how the various metal precursors react with the vacant sites of the framework, is essential, so as to permit the future synthesis of more uniform and more active materials.

### 3.2. Obtaining active-site homogeneity

**Figure 16.**
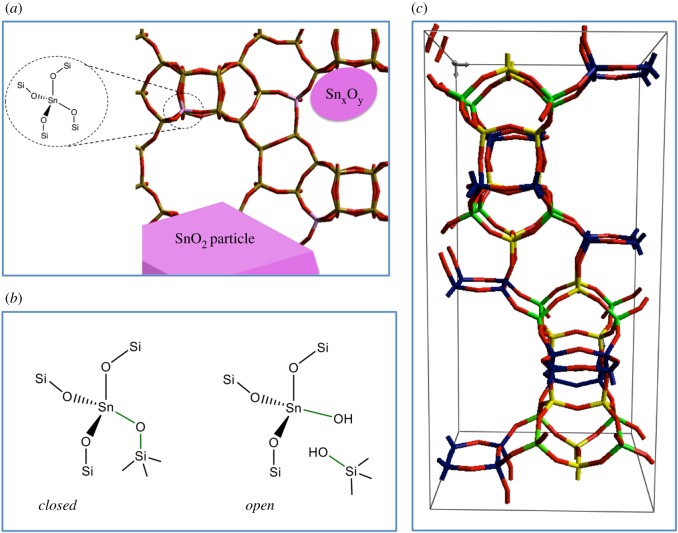
Various representations of the Sn sites potentially present in Sn-substituted zeolites. (*a*) Simplistic overview of framework and extra-framework Sn species. (*c*) The nine distinct crystallographic sites of the β-framework, designated T1–T9. Sites T1–T4 are blue; T5–T6 yellow and T7–T9 green. (*b*) Open and closed Sn sites, depending on the degree of framework hydrolysis. Reproduced with permission from Hammond [3].

Unlike their aluminosilicate counterparts, recent studies indicate that porous metallosilicates containing heteroatoms, such as Ti^4+^, Fe^3+^ and Sn^4+^, do not possess a uniform distribution of active sites. Indeed, it is becoming increasingly apparent that this class of catalyst typically contains a variety of active sites, only some of which may be involved in the catalytic chemistry of interest (figure 16). In addition to isomorphously substituted species, widely accepted to be responsible for catalytic performance in the case of Sn^4+^ and Ti^4+^, active sites in extra-framework positions of the material can also be formed. These can be isolated species simply bound to the framework, or larger nuclearity oligomers/clusters and bulk metal oxides [84,86]. Moreover, it should be noted that different framework sites exist, and in several types of zeolites there are up to 12 distinct framework positions within each unit cell (T-sites) [69]. Given that the precise T-site location of a particular heteroatom can dramatically modify its properties, preparing a catalyst with a different distribution of T-site species can remarkably influence the overall performance of the final catalyst [89]. In an added distinction, heteroatoms occupying identical T-sites can also exist in differing states of coordination and/or hydration. As such, identifying catalysts that exhibit uniform activity and/or selectivity, understanding how changing the active-site loading influences the final speciation of the catalyst and adequately characterizing such materials to develop accurate structure–activity–lifetime relationships, remains a formidable challenge.

Several consequences can arise from the presence of a mixed active site population. The first and most obvious is that the presence of species that do not contribute to the activity of the material results in a decreased catalytic performance, in terms of turnover frequency (mol. product/mol. active site). In addition to decreasing the atom efficiency of the catalyst, this can have important consequences with respect to intensification, by decreasing the space–time yield or catalytic productivity of the system. For example, we recently demonstrated that the turnover frequency of Sn-β decreased by 50% upon doubling the metal content of the catalyst from 5 wt% to 10 wt% [84]. Through a variety of spectroscopic methods, we demonstrated that this arises from the co-production of ‘spectator’ Sn sites (oligomeric and oxidic Sn species), which are inactive for the catalytic reaction of interest.

**Figure 17.**
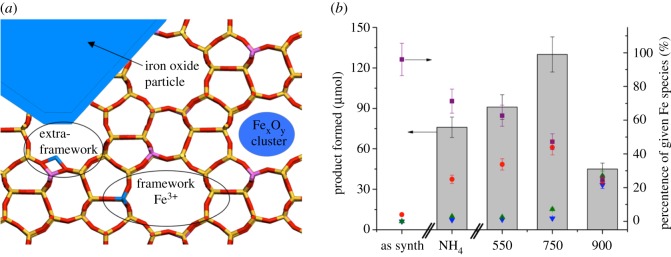
(*a*) Various Fe species identified in Fe-ZSM-5. (*b*) Correlation of catalytic activity of Fe-MFI zeolites for the oxidation of methane to methanol against the percentage of various Fe species absorbing at different wavelengths. Fe species absorbing between 250 and 350 nm (red circles) clearly correlate with performance. Reproduced with permission from Hammond *et al*. [86].

While in the case of Sn-β the presence of spectator species simply decreases the intrinsic performance of the catalyst, in other cases the presence of additional active sites can dramatically alter kinetic behaviour. For example, we previously demonstrated that Fe-containing zeolites, such as Fe-ZSM-5 possess a variety of active-site ensembles [82,86]. While detailed characterization of such materials is ongoing, initial analysis by UV-vis spectroscopy reveals that at least four types of Fe sites can be identified in such materials: (i) isomorphously substituted Fe^3+^ sites within the framework, (ii) electrostatically coordinated extra-framework Fe species in various oxidation states, (iii) oligomeric clusters of iron oxides (Fe_x_O_y_) and (iv) bulk iron oxide particles (figure 17*a*). The relative distribution of Fe among these species was found to be dependent on several factors, such as the method of synthesis, the Fe^3+^ and Al^3+^ loading of the catalyst, and the time and temperature of the heat treatment protocols [82,86]. In contrast with the case of Sn-β, however, kinetic studies reveal that the different Fe species are not simple spectators during the reaction of interest (catalytic conversion of methane to methanol with H_2_O_2_). In fact, several of the species were shown to be active for differing parts of the overall reaction network. For example, whereas our kinetic, spectroscopic and computational studies indicate that low-nuclearity, extra-framework Fe^3+^ species are probably responsible for selective activation of the H_2_O_2_ and methane [61,86], the same studies indicate that bulk iron oxide particles, present from progressive extraction of Fe^3+^ from the framework, are active for both the consecutive oxidation of methanol to formic acid and CO_x_, and the competitive (non-selective) decomposition of H_2_O_2_ (catalase reaction) [82,86]. In the case of Fe-ZSM-5, therefore, the presence of a mixed site distribution is not only detrimental to the overall productivity of the catalyst, but also decreases the selectivity of the reaction with respect to C and H_2_, with considerable amounts of relatively expensive oxidant being wasted. The simultaneous presence of different sites with different activities and selectivity also makes mechanistic studies especially challenging, although recent evidence indicates that a particular type of Fe site, as identified by UV-vis spectroscopy, correlates with catalytic activity (figure 17*b*).

**Figure 18.**
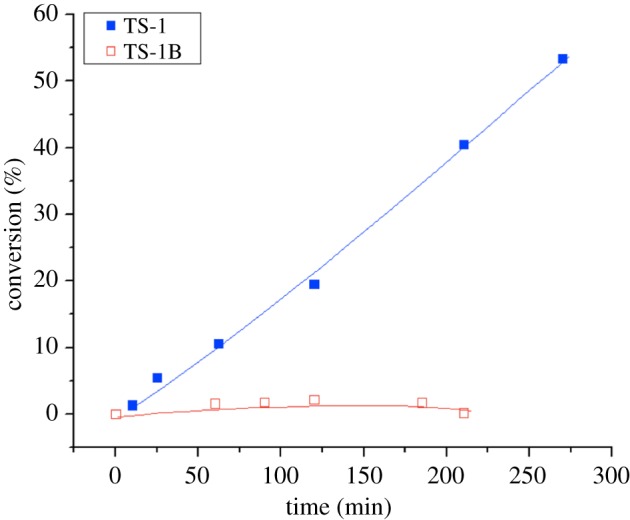
Conversion versus time for H_2_O_2_ decomposition at 60°C over TS-1 and NH_4_HF_2_-treated TS-1 (known as TS-1B). Reproduced with permission from Hammond & Tarantino [91].

Two important challenges arise from the phenomena of heterogeneous active-site distributions. The first challenge involves the development of methods by which the composition, and ultimately the performance, of these catalysts can be tailored to be more uniform. More uniform can imply more active on an activity per mole of active site basis, by removing spectator sites by decreasing the metal loading, or removing spectator sites by washing, as evidenced by van der Graaf *et al*. [90]. However, more uniform can also imply more selective to the desired reaction pathway, by minimizing side reactions and/or competitive reactions induced by alternative active sites. A key example of how this can be achieved can be highlighted from some of our team's recent research focused upon catalytic epoxidation with TS-1 [91]. Therein, we demonstrated that post-synthetic treatment of TS-1 with NH_4_HF_2_ and H_2_O_2_, following studies of Balducci *et al.* [92] resulted in the formation of a catalyst that was equally as active for catalytic epoxidation, but dramatically more efficient with respect to the oxidant, H_2_O_2_. Indeed, undesirable H_2_O_2_ decomposition was found to decrease almost entirely following this post-synthetic treatment (figure 18). The ability to switch off an undesirable side reaction, without majorly affecting the desired reaction pathway, clearly demonstrates that opportunities for maximizing the overall performance of various porous metallosilicates by optimizing their active-site distribution exist.

The second challenge that arises is the need to further develop spectroscopic methods capable of studying heterogeneous active-site distributions. Indeed, without the development of spectroscopic methods that are sufficiently sensitive, selective and quantitative towards the different types of active sites, developing accurate structure–activity–lifetime relationships will remain extremely challenging. Moreover, understanding how the active-site speciation changes as a function of synthesis protocol and catalytic operation will not be feasible.

**Figure 19.**
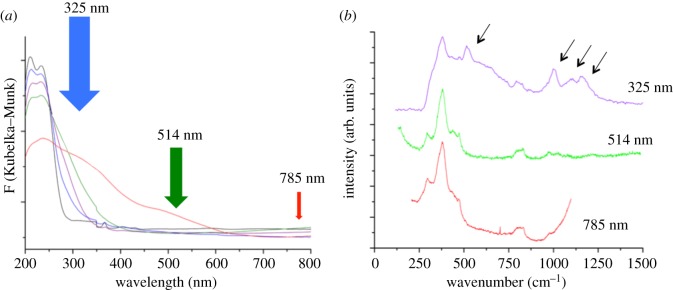
(*a*) Absorption spectra of various Fe-containing zeolites with heterogeneous active-site distributions, indicating the excitation lines that can be used to induce Raman scattering. (*b*) Raman spectra of Fe-containing zeolite collected at different excitation lines. Only by coinciding the excitation wavelength with the absorbing sites of the catalyst can detailed Raman bands of the active sites be observed (arrowed on the spectrum). Reproduced with permission from Hammond *et al*. [93].

Several techniques have been shown to be suitable for continued optimization. For example, probing the ligand-to-metal charge transfer bands (O^2− ^→ M^x+^) of various porous metallosilicates by UV-vis provides useful insight into the speciation of various metallosilicates, given that different active sites typically exhibit unique absorption features. For example, it has been demonstrated that a variety of active sites, including isomorphously substituted metal centres, oligomeric extra-framework species, and bulk oxides of Fe^3+^, Ti^4+^ and Sn^4+^, among others, can all be distinguished by simple absorption measurements of the catalyst (figure 19*a*) [86,87,90]. Particularly when combined with resonance Raman spectroscopy [93–96], substantial information regarding the speciation of the catalyst can be obtained (figure 19*b*). In contrast with other methodologies, such an approach allows the active sites to be followed selectively, by employing excitation wavelengths corresponding to a desired absorption band. For example, we recently demonstrated that collecting the Raman spectra of Fe-containing zeolites at an excitation wavelength of 325 nm allowed us to follow the vibrational spectra of the Fe^3+^ sites of the catalyst both during its synthesis, and following interaction with H_2_O_2_ [93]. Without coinciding the excitation wavelength with the absorption features of the active sites, no Raman features associated with Fe^3+^ could be observed. We note here that resonance Raman analysis of zeolites has recently experienced an explosion of interest, and readers interested in this aspect of characterization are directed to several of the works from the groups of Li and Solomon [97–99].

Other vibrational methods, such as FTIR, are also suitable for probing the active sites present within these materials, albeit indirectly through the adsorption of probe molecules. A variety of probe molecules, including Lewis bases (cyclohexanone, deuterated acetonitrile, propylamine and pyridine) and coordinating adsorbates such as CO and NO_x_, can be employed to study the active-site population of such materials [100–102]. A major advantage of this approach is that information on both the number and strength of active sites present in the sample can be obtained [103–105]. For example, Corma *et al.* were able to identify the presence of two types of isomorphously substituted Sn sites in zeolite *β* (closed and open, depending on the degree of framework hydrolysis) through the formation of two overlapping vibrational bands at 2308 and 2316 cm^−1^ following adsorption of *d*_3_-CD_3_CN onto the catalyst [103], although these assignments remain the topic of much debate [104]. The same team was also able to identify that the precise energy of this adduct vibration depends both on the identity of the heteroatom and the extent of substrate activation. For example, the activation of various carbonyl compounds was found to be substantially greater over Sn-containing zeolites (change in frequency of 48 cm^−1^) than for Ti- (32 cm^−1^), Zr- (40 cm^−1^) and Hf- (40 cm^−1^) containing zeolites [15]. Correlating the extent and degree of activation with catalytic performance, more quantitative structure–activity relationships can also be obtained. For example, Boronat *et al.* were able to correlate the activity of Sn-β for the Baeyer–Villiger oxidation of cyclic ketones to lactones to the number of hydrated Sn sites, based on a linear correlation between the number of open Sn sites (2316 cm^−1^) and the overall catalytic activity [103].

**Figure 20.**
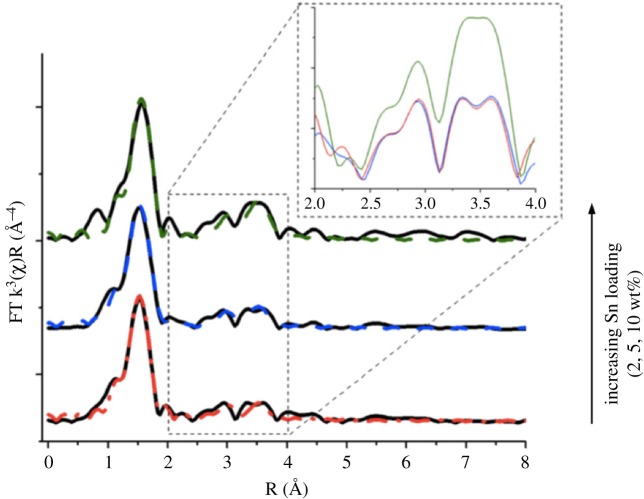
Increase in second shell (M-M) scattering as a function of Sn content in Sn-β prepared by SSI. Adapted with permission from Hammond *et al*. [84].

X-ray synchrotron methods, such as synchrotron XRD and X-ray adsorption spectroscopy (XAS), and magic angle spinning (MAS) NMR spectroscopy, have also shown promise. However, despite the excellent insights that can be obtained from XAS analysis, such methods have not yet been routinely employed to study these materials. The primary reasons for this include the fact that these methods require access to a synchrotron, prohibiting general utilization, and that XAS methods typically only provide averaged information on samples containing multiple active sites. However, with careful control they can prove to be useful. For example, we recently demonstrated that the relative amount of extra-framework Sn species present in Sn-β can readily be identified by extended X-ray absorption fine structure (EXAFS), by comparing the relative intensities of the first (M-O) and second shell (M-M) features. By using this ratio, we were able to quantify the relative amount of extra-framework Sn sites present in the material following solid-state incorporation of Sn at different Sn loadings (figure 20) [84]. This analysis revealed that while catalytically active, isomorphously substituted Sn^4+^ sites dominate the active-site population up to 5 wt% Sn, extra-framework Sn^4+^ oligomers are produced at higher Sn loadings. Support for these observations was also obtained by ^119^Sn MAS NMR analysis of the samples (vide infra).

Unfortunately, such methods are unable, as yet, to provide a thorough discrimination among heteroatoms present in different T-sites of the framework. However, as previously described, the precise T-site occupied by the heteroatom of interest can profoundly impact catalytic performance, given that each T-site represents a slightly different molecular environment. Consequently, determining the T-site occupation of various heteroatoms is a challenge of immense importance [106]. Among recent breakthroughs, Bare *et al.* demonstrated EXAFS can provide insight into the T-site occupation of metallosilicates, following advanced fitting protocols of the higher shell data [69]. In doing so, the authors proposed that Sn preferentially occupies the T5/T6 sites of the *β* framework, and that it preferentially substitutes in pairs on opposite sides of the same rings. Similar studies focused on the T-site occupation of Ti- or Al-containing MFI zeolites have also been performed with synchrotron XRD, X-ray standing wave, neutron diffraction and conventional EXAFS [106–109]. However, to date, multiple conclusions have been obtained for identical catalytic materials with each of these methods suggesting that further research and progress is still required with these methods. More uniform insights regarding T-site occupation have, however, been obtained by MAS NMR, although the technique is evidently only selective to NMR-active nuclei, such as ^119^Sn. Among recent studies, greatest insight has been achieved when hyperpolarization methods are employed, such as DNP-MAS NMR [110,111]. In this case, exogenous biradicals and microwave radiation are used to transfer polarization to the Sn sites, allowing spectra to be collected with several order of magnitude increases in the signal-to-noise ratio. Although the presence of biradical species may influence the spectroscopic results achieved, recent studies with CPMG MAS NMR indicate that comparable data can be achieved even in the absence of exogeneous radical initiators [112]. An excellent example of how such techniques can be used to study porous metallosilicates was recently reported by Wolf *et al.* Therein, DNP-MAS NMR was used to study Sn-β samples with cross-polarization magic-angle turning (CPMAT), in order to extract information typically hidden in the chemical shift anisotropy (CSA) [113]. Given that the CSA varies between T-sites, analysing this information allowed the group to determine the preferential T-site occupations of stannosilicate materials prepared by various methods.

Despite this progress, however, several hurdles still limit the opportunities for researchers to accurately study these materials with spectroscopic methods. For example, several of the methods are not generally applicable, being only suitable to specific materials (e.g. ^119^Sn MAS NMR), or requiring advanced infrastructure (e.g. synchrotron). As such, benchmark and universal studies of various materials cannot yet be performed. Finally, it should not be forgotten that characterization of the active sites of these materials should, preferably, be undertaken in the true reaction environment. As such, perhaps the major challenge in this area includes the development of techniques that are selective and sensitive to the active sites, even when the catalyst is in a liquid medium. Unfortunately, most methods to date have studied the dehydrated form of such materials outside of the catalytic reactor, and hence the insights achieved to date may not be totally representative of the real catalytic materials. Indeed, in the presence of the additional species (reactants/products/solvent molecules) the speciation of the active sites may be altered significantly.

### 3.3. Intensification studies and continuous operation

In addition to possessing high levels of catalytic activity and excellent levels of target product selectivity, promising heterogeneous catalysts also need to demonstrate excellent levels of stability [114]. In fact, determining the ability of the catalyst to be used without loss in performance for a sufficient period of time is one of the most important targets. Given the novel nature of the porous metallosilicates discussed above, in addition to the novel types of catalytic chemistry they mediate, identifying and optimizing the stability of such materials represents a formidable challenge.

**Figure 21.**
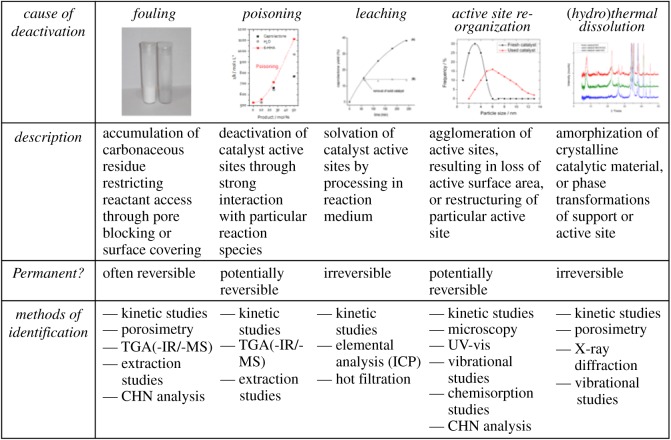
Overview to the type of deactivation events experienced by heterogeneous catalysts during liquid-phase operation, along with methods of detection. Reproduced with permission from Hammond [115].

Several possible mechanisms and events can lead to deactivation of porous metallosilicate catalysts (figure 21). Generally, these processes can be grouped into two categories, those being ‘reversible’ and ‘irreversible’ [114,115]. In the former, deactivation is not permanent, and further processing of the catalyst can restore activity. In the second case, deactivation is permanent, resulting in total loss of the catalyst. Although avoiding irreversible deactivation is evidently critical, maximizing the time a catalyst can operate for without requiring regeneration is also essential, as such steps lead to unwanted downtime and additional processing costs, and can place undesirable requirements on the overall reactor design [116]. Accordingly, identifying the cause(s) of deactivation, and devising strategies to minimize its impact, are critical areas of catalysis research. However, several factors complicate this challenge. For example, the deactivation events outlined in figure 21 can often operate in parallel, working collaboratively to contribute to a global loss in activity. Moreover, the identification of the causes of deactivation often requires advanced spectroscopic study of the catalytic materials pre- and post-operation. Accordingly, several of the spectroscopic hurdles outlined contribute. As TS-1 already operates industrially, it is clear that porous metallosilicates can be tailored to be sufficiently stable for continuous operation on an industrial level. However, comparable studies of other porous metallosilicates are scarce or non-existent. Given that these other materials exhibit different properties to TS-1, and catalyse very different types of reactions, it is not yet clear that they possess sufficient levels of stability for industrial operation.

**Figure 22.**
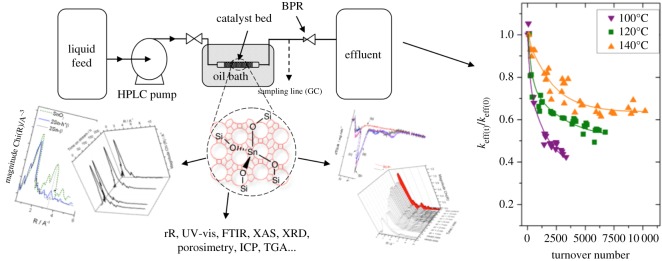
Schematic overview of our method, combining detailed kinetic studies of catalytic materials in scalable continuous reactors, with advanced spectroscopic study of the catalyst pre- and post-operation.

In contrast with conventional recyclability studies, our studies in this area employ a more rigorous evaluation of stability [117–121], by performing kinetic studies in continuous plug flow reactors. Such reactors (figure 22) provide several advantages over batch reactors, including (i) improved process and safety control, (ii) higher levels of mass and heat transfer, (iii) shorter reaction times, (iv) minimized reactor volumes, (iv) higher levels of scalability and (v) improved space–time yields. Additionally, such reactors permit steady-state operation, allowing catalyst deactivation to be probed under industrially relevant conditions. Particularly when combined with spectroscopic characterization methodologies, which allow the physico-chemical properties of the material pre- and post-reaction to be characterized, such an approach is a powerful and accurate method to rigorously assess catalyst stability.

**Figure 23.**
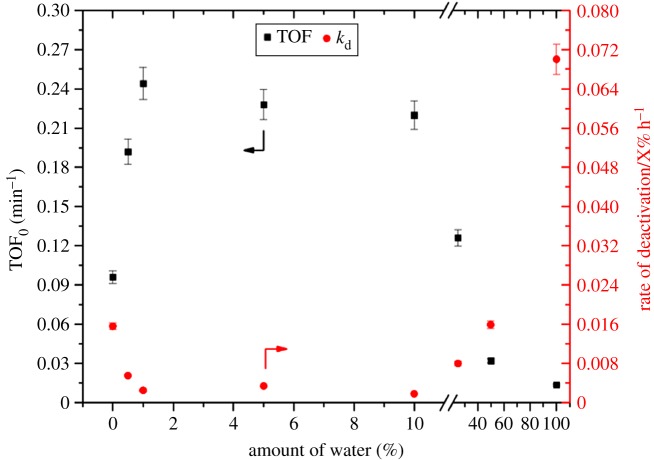
Rate of deactivation of Sn-β for the isomerization of glucose to fructose (right *y*-axis), along with the TOF of the catalyst (left *y*-axis) at various solvent compositions, ranging from 100% methanol to 100% water. Reproduced with permission from Padovan *et al*. [117].

These recent studies have highlighted several important factors that influence the stability of Sn-β during operation [118–121]. The most critical factor influencing stability of such materials is the choice of reaction solvent. In addition to influencing the extent of deactivation, the choice of solvent can even impact the mechanism of deactivation. For example, we recently demonstrated, through spectroscopic studies, that irreversible deactivation of Sn-β for the isomerization of glucose to fructose occurred in water through amorphization of the zeolite [118]. By contrast, performing the reaction in methanol led to a shift in deactivation mechanism, with fouling being found to be responsible for the decrease in activity. Notably, fouling is reversible, and periodic regeneration of the catalyst bed was found to restore full levels of activity. It is therefore clear that despite its apparent water tolerance, an aqueous medium is far from ideal for continuous Sn-β catalysis [122]. Curiously, our most recent study indicates that despite water itself being a very unfavourable solvent for continuous operation of Sn-β, adding a small amount of water to the methanol solvent leads to dramatic improvements in catalyst stability (figure 23) [117]. Indeed, the rate of deactivation experienced by Sn-β decreased by over one order of magnitude, upon the addition of between 1 and 10 wt% water to methanol. Notably, catalytic performance per quantity of Sn also improved by a factor of 2.5 following the addition of water. Clearly, optimizing the overall reaction system, and particularly the choice of solvent, can lead to dramatic improvements in overall performance.

We have also observed that the precise choice of reaction conditions can also affect the stability of the catalyst. For example, we recently observed that the deactivation of Sn-β for the catalytic transfer hydrogenation of cyclohexanone could be minimized substantially, increasing the reaction temperature from 110 to 140°C [118]. Through thermogravimetric and NMR analysis, this improvement was attributed to improved desorption of the reaction product, cyclohexanol, which we found to be susceptible to polymerization in the pores of the zeolite, resulting in pore fouling.

**Figure 24.**
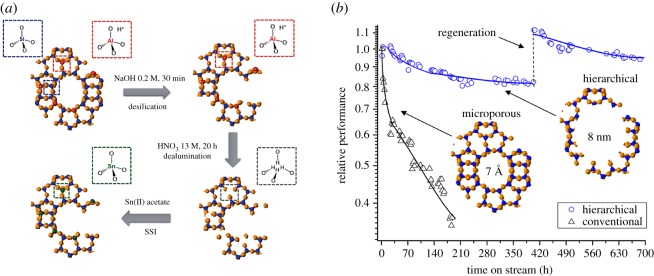
(*a*) Procedure for the preparation of hierarchical Sn-β. (*b*) Relative performance of microporous (triangles) and hierarchical (circles) Sn-β during the CTH of cyclohexanone, along with indicative pore sizes of the materials. Figures adapted with permission from Al-Nayili *et al*. [120].

However, while optimizing the processing conditions can dramatically improve catalyst longevity, this strategy does not improve the intrinsic stability of the catalytic material. Accordingly, catalyst design and modification strategies can also be investigated in an effort to improve long-term stability. For example, we recently demonstrated that modifying the pore structure of metallosilicates can dramatically improve their continuous performance. Specifically, by converting a purely microporous stannosilicate to a hierarchical analogue, which possesses a combination of both micropores and mesopores, we were able to minimize the rate of deactivation by over one order of magnitude (figure 24*b*) [120]. The improved performance in this case can be attributed to the co-presence of mesopores in the structure, which minimize the propensity of the material to foul through pore blocking [90]. To convert the microporous material to the hierarchical analogue, partial desilication of the framework was achieved by treatment in dilute NaOH (figure 24*a*). While this treatment did not modify the intrinsic kinetic behaviour of the material, catalyst stability improved by one order of magnitude; over a period of 700 h on stream, hierarchical Sn-β was found to lose only approximately 20% of its maximal performance, whereas the microporous analogue lost more than 60% of its activity in less than 200 h. This study further demonstrates that relatively subtle changes to the composition and/or structure of the metallosilicate can result in dramatic improvements in overall performance. However, while several studies have focused upon the generation of novel materials containing unique types of active sites, very few studies have focused upon the design of materials that are intrinsically more stable. The development of such materials therefore represents a major challenge for future studies.

## 4. Conclusion

Porous silicates containing heteroatomic sites of Ti, Sn and Fe, among others, are clearly extremely promising catalysts for heterogeneous, liquid-phase catalytic technologies. Their unique properties, and particularly their tuneable molecular-sieving and acid–base–redox properties, have resulted in them displaying unique reactivity patterns for a range of emerging catalytic transformations, including the conversion of biomass to chemicals, and selective oxidation processes with the green oxidant, H_2_O_2_. However, continued utilization of these materials, especially at greater scale, clearly depends on further progress being achieved in several areas. Some of the most important pertaining challenges, highlighted throughout this review, include (i) the development of improved, and more scalable, material preparation methodologies; (ii) the ability for researchers to control the active-site distribution, and obtain more uniform catalysts; (iii) the ability to study the speciation of these heterogeneous catalysts with selective and quantitative spectroscopic methods; (iv) the development of catalytic materials and processes more amenable to continuous operation. Moreover, (v) the continued development of novel reactions, especially related to sustainability, remains an ever-present challenge. These challenges therefore represent the key targets that we, and others, aim to solve in the forthcoming years.

## Data accessibility

As a review article, no original data are included in the article, and hence no original datasets are available.
